# LAsting Symptoms after Oesophageal Resectional Surgery (LASORS): multicentre validation cohort study

**DOI:** 10.1093/bjs/znae319

**Published:** 2025-02-21

**Authors:** Heidi Paine, Swathikan Chidambaram, Asif Johar, Nick Maynard, Pernilla Lagergren, Ewen A Griffiths, Paul Behrens, Pritam Singh, Nima Abbassi-Ghadi, Shaun R Preston, Ravinder S Vohra, James Gossage, Tim Underwood, Nick Dai, J Robert O’Neill, Sherif Awad, Borzoueh Mohammadi, Khaled Dawas, Yassar Qureshi, Bilal Alkhaffaf, Rhys Jones, George B Hanna, Sheraz R Markar

**Affiliations:** Nuffield Department of Surgery, University of Oxford, Oxford, UK; Department of Surgery, Churchill Hospital, Oxford University Hospitals NHS Trust, Oxford, UK; Academic Surgical Unit, Department of Surgery and Cancer, Imperial College London, St Mary’s Hospital, London, UK; Department of Molecular Medicine and Surgery, Karolinska Institutet, Stockholm, Sweden; Department of Surgery, Churchill Hospital, Oxford University Hospitals NHS Trust, Oxford, UK; Academic Surgical Unit, Department of Surgery and Cancer, Imperial College London, St Mary’s Hospital, London, UK; Department of Molecular Medicine and Surgery, Karolinska Institutet, Stockholm, Sweden; Department of Upper GI Surgery, University Hospitals Birmingham NHS Foundation Trust, Birmingham, UK; Edinburgh Law School, University of Edinburgh, Edinburgh, UK; Department of Surgery, Royal Surrey County Hospital NHS Foundation Trust, Guildford, UK; Department of Surgery, Royal Surrey County Hospital NHS Foundation Trust, Guildford, UK; Department of Surgery, Royal Surrey County Hospital NHS Foundation Trust, Guildford, UK; Department of Surgery, Nottingham University Hospitals NHS Trust, Nottingham, UK; Department of Surgery, St Thomas’ Hospital, London, UK; Division of Surgery, University Hospital Southampton NHS Foundation Trust, Southampton, UK; Cambridge Oesophagogastric Centre, Cambridge University Hospitals, Cambridge, UK; Cambridge Oesophagogastric Centre, Cambridge University Hospitals, Cambridge, UK; Edinburgh Cancer Research, Institute of Genetics and Cancer, University of Edinburgh, Edinburgh, UK; Department of Upper GI Surgery, Royal Derby Hospital, University Hospitals of Derby & Burton NHS Foundation Trust, Derby, UK; Department of Surgery, University College London Hospitals NHS Foundation Trust, London, UK; Department of Surgery, University College London Hospitals NHS Foundation Trust, London, UK; Department of Surgery, University College London Hospitals NHS Foundation Trust, London, UK; Department of Oesophago-Gastric & Bariatric Surgery, Salford Royal Hospital, Northern Care Alliance NHS Foundation Trust, Manchester, UK; Division of Cancer Sciences, School of Medical Sciences, Faculty of Biology, Medicine and Health, University of Manchester, Manchester, UK; Department of Surgery, James Cook University Hospital, Middlesborough, UK; Academic Surgical Unit, Department of Surgery and Cancer, Imperial College London, St Mary’s Hospital, London, UK; Nuffield Department of Surgery, University of Oxford, Oxford, UK; Department of Surgery, Churchill Hospital, Oxford University Hospitals NHS Trust, Oxford, UK

## Abstract

**Background:**

Long-term symptom burden and health-related quality-of-life outcomes after curative oesophageal cancer treatment are poorly understood. Existing tools are cumbersome and do not address the post-treatment population specifically. The aim of this study was to validate the six-symptom LASORS tool for identifying patients after curative oesophageal cancer treatment with poor health-related quality of life and to assess its clinical utility.

**Methods:**

Between 2015 and 2019, patients from 15 UK centres who underwent curative-intent oesophageal cancer treatment, and were disease-free at least 1 year after surgery, were invited to participate in the study and complete LASORS and European Organisation for Research and Treatment of Cancer QLQ-C30 and QLQ-OG25 questionnaires. Receiver operating characteristic curve analysis was used to examine the accuracy of the LASORS tool for identifying patients with poor health-related quality of life.

**Results:**

A total of 263 patients completed the questionnaire. Four of the six LASORS symptoms were associated with poor health-related quality of life: reduced energy (OR 2.13 (95% c.i. 1.45 to 3.13)); low mood (OR 1.86 (95% c.i. 1.20 to 2.88)); diarrhoea more than three times a day unrelated to eating (OR 1.48 (95% c.i. 1.06 to 2.07)); and bloating or cramping after eating (OR 1.35 (95% c.i. 1.03 to 1.77)). The LASORS tool showed good diagnostic accuracy with an area under the receiver operating characteristic curve of 0.858 for identifying patients with poor health-related quality of life.

**Conclusion:**

The six-symptom LASORS tool generated a reliable model for identification of patients with poor health-related quality of life after curative treatment for oesophageal cancer. This is the first tool of its kind to be prospectively validated in the post-esophagectomy population. Clinical utility lies in identification of patients at risk of poor health-related quality of life, ease of use of the tool, and in planning survivorship services.

## Teaser

This study is the culmination of the previously published LASER study research, which identified a core set of symptoms associated with poor health-related quality of life. In this study the authors went on and prospectively validated the six-symptom LASORS tool in a UK cohort of patients and demonstrated good accuracy with an area under the receiver operating characteristic curve of 0.858. The importance of this study is that it provides validation of the LASORS tool, which can potentially be used in clinical practice to guide follow-up of patients after oesophagectomy with more appropriate allocation to survivorship clinics.

## Introduction

Oesophageal cancer is the 11th most common cancer worldwide and the 7th most common cause of cancer-related mortality^1–3^. Recent advances in oncological therapies and surgical techniques, as well as centralization of services and concentration of expertise, have produced significant improvements in oncological outcomes and survival in recent years^4,5^. Patients must then live with the medium-to-long-term physical and psychosocial effects of their cancer treatment, with one population-based cohort study suggesting 40% of patients seek medical attention for long-term symptoms post-oesophagectomy, with a strong correlation between symptom burden and new onset of anxiety and depression after surgery^6^. To meet the needs of this expanding cohort of patients, treatment must encompass the long-term survivorship implications and services must be made available to address the specific challenges.

With increasing recognition of the long-term symptom burden imposed on patients who have undergone an oesophagectomy, existing tools, such as the European Organisation for Research and Treatment of Cancer (EORTC) health-related quality of life (HRQoL) tools, have been used in the context of oesophageal cancer research^7^. However, these questionnaires are too time-consuming and cumbersome to be used routinely in clinical practice. Moreover, they were designed to be used for, and are validated in, patients undergoing treatment, rather than for the cancer-free survivorship cohort, whose nature of symptoms may differ.

Surveillance after curative oesophageal cancer treatment varies widely across units, regions, and countries, and often focuses largely on oncological parameters, rather than functional and psychosocial outcomes. Moreover, where long-term symptoms are recognized, infrastructure and access to expertise to address these are often lacking. Detection and appropriate management of these symptoms is paramount and their cumulative impact becomes increasingly important and burdensome for patients as cancer-free survival improves. Therefore, there is a need for a tool that is simple to administer, specific to the disease of oesophageal cancer, and validated in the post-treatment population.

Through a multicentre European study, the authors have previously identified three key symptoms that were independently associated with poor HRQoL, as measured using validated EORTC tools^8^. After consultation with clinicians in conjunction with the Oesophageal Patient Association (UK) and Heartburn Cancer UK patient support groups, three further symptoms were identified and combined with those from the LASER study, forming the LAsting Symptoms after Oesophageal Resectional Surgery (LASORS) clinical symptom tool^9^. The aim of this study was to validate this six-symptom clinical tool for identifying patients with poor HRQoL identified using EORTC QLQ-C30 and QLQ-OG25 questionnaires, at least 1 year after curative-intent surgery for oesophageal cancer.

## Methods

### Study design and participants

**Fig. 1:**
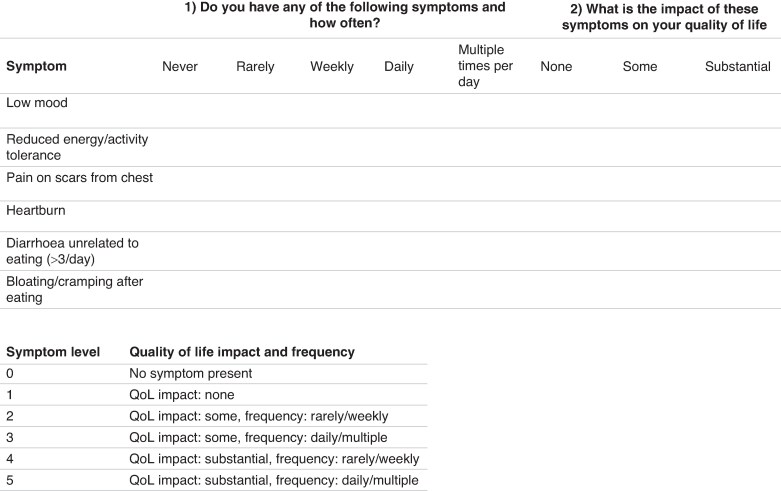
LASORS tool Symptom-based grading system including prevalence and impact on health-related quality of life; each symptom is then awarded a composite score from zero to five.

The authors conducted a prospective cohort study to validate the LASORS tool for identifying patients with poor HRQoL after oesophagectomy. Patients who were disease-free 1 year after surgery were screened for inclusion. Eligible patients from high-volume centres in the UK were invited to complete the LASORS questionnaire, as well as EORTC QLQ-C30 and QLQ-OG25 questionnaires. Centres were contacted through the Association of Upper Gastrointestinal Surgery of Great Britain and Ireland (AUGIS) research network. The inclusion criteria for this study were: patients aged over 18 years at the time of surgery; individuals who had undergone oesophagectomy for oesophageal or gastro-oesophageal junctional cancer (Siewert I and II) between January 2015 and June 2019; and patients who had completed cancer treatment, whether surgical or oncological, at least 1 year ago. The authors excluded any patient who was unable to provide informed consent and any patient with evidence of cancer recurrence, as detected by local centres based on their routine follow-up protocols. Patients completed the LASORS questionnaire (*Fig. 1*).

### Outcome measures

Each symptom from the LASORS questionnaire was graded based on its impact on HRQoL and its frequency, yielding a composite score ranging from zero to five. The EORTC HRQoL symptom items included four categories on a Likert scale: 1, not at all; 2, a little; 3, quite a bit; and 4, very much. The LASORS symptom tool was validated against poor/good HRQoL, as measured using EORTC QLQ-C30 and QLQ-OG25 questionnaires. Poor HRQoL was defined as having poor function and poor symptoms according to the QLQ-C30 and QLQ-OG25 questionnaires, indicated by answering ‘quite a bit’ or ‘very much’ to at least one question in both the function and symptom scales. Patients who did not meet this criterion were considered to have good HRQoL.

Demographic data, such as patient age, sex, BMI, ethnic background, socio-economic status (Carstairs index), education level, smoking status, and medical co-morbidities (collated using the Charlson co-morbidity index), were obtained through a review of medical records. Details of surgery including tumour stage, location, type of surgery, and the use of neoadjuvant and adjuvant therapies, as well as specific data on postoperative complications (as defined by the Esophageal Complications Consensus Group) and long-term complications, were collected by reviewing medical records.

### Questionnaires

The EORTC QLQ-C30 questionnaire is a standardized questionnaire designed to assess the QoL of cancer patients. It consists of 30 items with both multi-item scales and single-item measures. It covers five functional scales (physical, role, emotional, cognitive, and social), three symptom scales (fatigue, nausea and vomiting, and pain), a global health status/QoL scale, and items assessing additional symptoms reported by cancer patients, as well as perceived financial impact of the disease and treatment​. Each item is rated on a Likert scale, typically with responses ranging from ‘not at all’ to ‘very much’ for symptom scales and from ‘very poor’ to ‘excellent’ for the global health status/QoL scale. The scores from these scales and single items provide a comprehensive picture of a patient’s QoL.

The authors described the development of the LASORS tool in their earlier work. Briefly, the authors’ previous work involved a systematic review of the literature to identify common symptoms experienced by patients, which were then finalized through a Delphi consensus meeting. Based on multivariable analysis of self-reported symptom and HRQoL ratings from 876 patients, the authors generated three symptoms that correlated with poor HRQoL: pain at the thoracic incision site; low mood; and reduced energy. In addition, the authors included three further symptoms (heartburn; diarrhoea more than 3 times a day unrelated to eating; and bloating or cramping after eating) after consultation with their patient group representatives. The LASORS tool first measures the frequency of these symptoms using the options ‘never’, ‘rarely’, ‘weekly’, ‘daily’, and ‘multiple times per day’. It subsequently assesses the impact of the symptoms on HRQoL using the options ‘none’, ‘some’, and ‘substantial’.

### Patient and public involvement

The findings of the authors’ previous study were presented to the Oesophageal Patient Association (UK) and Heartburn Cancer UK. The authors invited patient representatives from these groups to be on the steering committee. The study development incorporated their input during various patient and public involvement workshops.

### Statistical methodology

A sample size of 640 patients was calculated to validate this tool, aiming for a sensitivity and specificity of 80%. Anticipating a response rate of 80%, 800 patients were required for recruitment. This was based on simple nomograms and a predicted prevalence of poor HRQoL of 45% for the previously developed LASORS clinical symptom tool. An interim analysis was planned after 250 patients to assess futility of further recruitment.

Each HRQoL symptom item comprised four categories on a Likert scale: 1, not at all; 2, a little; 3, quite a bit; and 4, very much. Linear transformation of Likert scores was performed based on EORTC guidance to a numerical value of 0–100, with higher scores indicating more severe symptoms. Each symptom from the LASORS questionnaire was assessed based on its impact on HRQoL and its frequency, resulting in a composite score ranging from zero to five. The area under the receiver operating characteristic (ROC) curve was used to measure the overall accuracy of the prediction model for test and validation cohorts, which were generated through random allocation from the entire data set. All statistical analyses were performed by an experienced biostatistician (A.J.) using SAS 9.4 (SAS institute Inc., Cary, NC, USA).

## Results

In total, 322 patients were invited to participate from 15 UK centres and 263 participants were included in the study (a response rate of 82%) (*Table S1*).

### Patient characteristics and health-related quality of life, as measured using EORTC tools

*Table 1* shows the frequency of poor HRQoL according to baseline demographics and treatment characteristics. Poor QoL was more common in females than males when stratified by sex (54.2% *versus* 44.8% respectively). Patients with an ASA grade of at least II were more likely to report poor HRQoL compared with those with an ASA grade of I.

**Table 1:** Summary of frequency of poor and good health-related quality of life ratings according to patient, treatment, and postoperative characteristics

Category	Frequency	Good HRQoL	Poor HRQoL
**Sex**			
Male	192 (76.5)	106 (55.2)	86 (44.8)
Female	59 (23.5)	27 (45.8)	32 (54.2)
**ASA grade**			
I	38 (15.0)	21 (55.3)	17 (44.7)
II	158 (62.2)	90 (57.0)	68 (43.0)
III	57 (22.4)	25 (43.9)	32 (56.1)
IV	1 (<1)	0 (0.0)	1 (100.0)
**Pathological stage**			
0	14 (5.5)	8 (57.1)	6 (42.9)
I	67 (26.2)	38 (56.7)	29 (43.3)
II	89 (34.8)	52 (58.4)	37 (41.6)
III	82 (32.0)	37 (45.1)	45 (54.9)
IV	4 (1.6)	2 (50.0)	2 (50.0)
**Surgical access**			
Hybrid minimally invasive (laparoscopy and open thoracotomy)	36 (14.0)	18 (50.0)	18 (50.0)
Hybrid minimally invasive (open abdomen and thoracoscopic chest)	10 (3.9)	6 (60.0)	4 (40.0)
Totally minimally invasive (laparoscopic abdomen and thoracoscopic chest)	54 (21.0)	26 (48.1)	28 (51.9)
Totally minimally invasive (robotic abdomen and chest)	1 (<1)	0 (0.0)	1 (100.0)
Totally open (open abdomen and thoracotomy)	156 (60.7)	86 (55.1)	70 (44.9)
**Surgical technique**			
Ivor Lewis (2-stage)	183 (70.9)	90 (49.2)	93 (50.8)
Left thoraco-abdominal	36 (14.0)	25 (69.4)	11 (30.6)
McKeown (3-stage)	38 (14.7)	21 (55.3)	17 (44.7)
Transhiatal	1 (<1)	0 (0)	1 (100)
**Anastomosis location**			
Cervical	38 (15.1)	21 (55.3)	17 (44.7)
Thoracic	213 (84.9)	113 (53.1)	100 (46.9)
**Neoadjuvant therapies**			
Chemotherapy	144 (57.4)	85 (59.0)	59 (41.0)
Chemoradiotherapy	59 (23.5)	26 (44.1)	33 (55.9)
None	48 (19.1)	22 (45.8)	26 (54.2)
**Postoperative complications**			
No	113 (43.3)	65 (57.5)	48 (42.5)
Yes	148 (56.7)	74 (50.0)	74 (50.0)

Values are *n* (%). HRQoL, health-related quality of life.

With respect to surgical access, patients in the hybrid minimally invasive (open abdomen and thoracoscopic chest) group and the totally open (open abdomen and thoracotomy) group were more likely to have good than poor HRQoL; the reverse was true of those in the totally minimally invasive (robotic abdomen and chest) group and the totally minimally invasive (laparoscopic abdomen and thoracoscopic chest) group (100% and 52% poor HRQoL respectively). Patients who underwent open left thoraco-abdominal and McKeown oesophagectomy were more likely to have good than poor HRQoL scores (31% and 45% poor HRQoL respectively), with the reverse seen in the Ivor Lewis group and the transhiatal group (51% and 100% poor HRQoL respectively).

Stage of cancer had an impact on HRQoL scores, with patients with stage 0, I, and II cancer more likely to report good than poor HRQoL (43%, 43%, and 42% poor HRQoL respectively). Patients with stage III cancer were more likely to report poor than good HRQoL (55% poor HRQoL).

Patients who underwent chemotherapy were more likely to report good than poor HRQoL, in contrast to those undergoing chemoradiotherapy or no neoadjuvant treatment (41% *versus* 56% *versus* 54% poor HRQoL respectively).

Patients who experienced postoperative complications were more likely to report poor HRQoL compared with those who did not.

### Association between LASORS symptoms and health-related quality of life

**Fig. 2:**
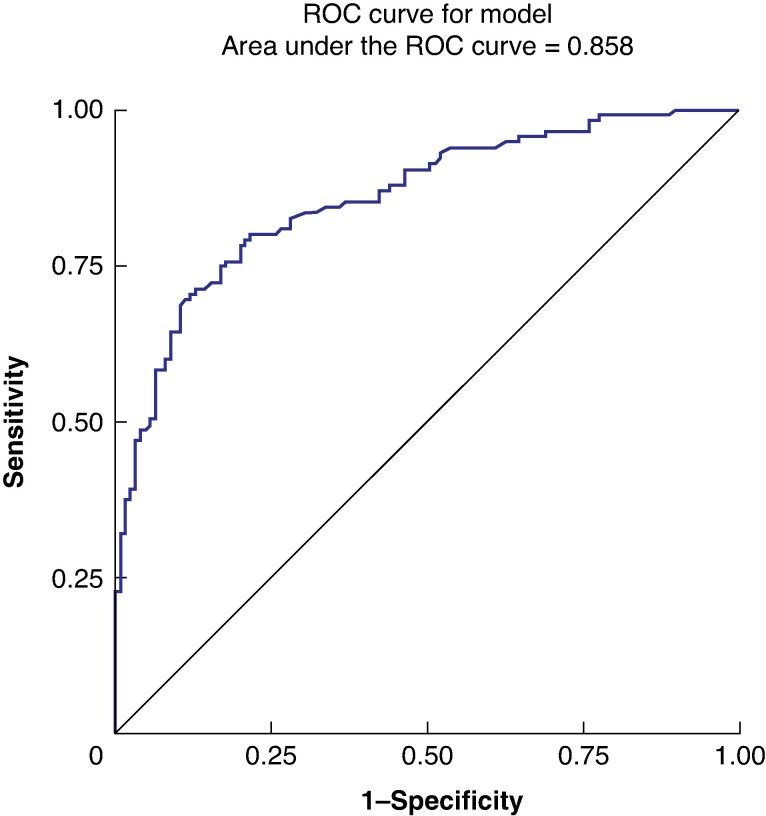
Receiver operating characteristic curve for LASORS symptoms and health-related quality of life

Four LASORS symptoms were associated with significantly lower HRQoL: reduced energy (OR 2.13 (95% c.i. 1.45 to 3.13)); low mood (OR 1.86 (95% c.i. 1.20 to 2.88)); diarrhoea more than three times a day unrelated to eating (OR 1.48 (95% c.i. 1.06 to 2.07)); and bloating or cramping after eating (OR 1.35 (95% c.i. 1.03 to 1.77)) (*Table 2*). Although the HRQoL was lower in patients reporting pain at the thoracic incision site (OR 1.01 (95% c.i. 0.67 to 1.54)) and heartburn (OR 1.10 (95% c.i. 0.83 to 1.45)), this was not statistically significant. The area under the ROC curve was 0.858 for all six LASORS symptoms combined (*Fig. 2*). Overall, the sensitivity was 0.821 (95% c.i. 0.76 to 0.89), the specificity was 0.731 (95% c.i. 0.65 to 0.81), the positive predictive value was 0.78 (95% c.i. 0.71 to 0.84), and the negative predictive value was 0.78 (95% c.i. 0.71 to 0.86).

ROC, receiver operating characteristic.

**Table 2:** Summary of ORs for LASORS symptoms and health-related quality of life

Symptom	OR (95% c.i.)
**Low mood**	1.86 (1.20,2.88)
**Reduced energy**	2.13 (1.45,3.13)
**Pain at the thoracic incision site**	1.01 (0.67,1.54)
**Heartburn**	1.10 (0.83,1.45)
**Diarrhoea >3 times a day unrelated to eating**	1.48 (1.06,2.07)
**Bloating or cramping after eating**	1.35 (1.03,1.77)

An additional analysis was performed where ‘poor HRQoL’ was defined as answering ‘quite a bit’ or ‘very much’ to either a function or symptom question (rather than in both domains, as above). The LASORS tool predicted poor HRQoL with an area under the ROC curve of 0.858, with a sensitivity and specificity of 0.821 and 0.731 respectively.

## Discussion

This multicentre UK study of 263 patients validates the previous LASER work and the clinical LASORS tool for predicting patients with poor HRQoL based upon EORTC questionnaires. Four of the six LASORS symptoms independently predicted poor HRQoL (reduced energy, low mood, diarrhoea more than 3 times a day unrelated to eating, and bloating or cramping after eating), with pain at the thoracic incision site and heartburn showing non-significant trends towards poor HRQoL. The area under the ROC curve was 0.858 for the LASORS tool. Recruitment was terminated early at 263 patients, due to validation of the LASORS tool through this futility analysis and demonstration of clear accuracy of the tool.

Overall, 46% of patients reported poor HRQoL using the LASORS questionnaire. Patients with postoperative complications were more likely than those without postoperative complications to report poor HRQoL, highlighting the importance of striving to reduce the frequency and impact of postoperative complications. Interestingly, an open approach was associated with a lower rate of poor HRQoL, building on previous findings from the senior author's group that demonstrated no beneficial impact of a minimally invasive surgical technique on HRQoL^10^ and from the ROMIO study that reported no benefit of a hybrid over an open approach with regard to physical functioning at 3 months^11^. These findings, in the context of minimally invasive oesophagectomy having been shown to reduce short-term postoperative complications^12–14^, suggest that focus should be not just on mitigating immediate perioperative complications but also on mitigating medium-to-long-term postoperative complications.

Reduced energy, diarrhoea more than three times a day unrelated to eating, and bloating or cramping after eating are among the most prevalent sequelae of oesophagectomy^8,15^ and the authors’ finding that they independently predict poor HRQoL should prompt further research into the anatomical and physiological mechanisms underpinning them^16^. One of the symptoms correlating most strongly with poor HRQoL was low mood. Psychological well-being has been shown to be important post-oesophagectomy, with national cohort studies suggesting poorer overall survival in patients diagnosed with depression after oesophagectomy^17,18^. Therefore, it is important to consider not just the physical and functional sequelae of surgery but also the psychological impact of cancer treatment.

In the present study, pain at the thoracic incision site was not independently associated with poor HRQoL, in contrast to the findings of studies in thoracic surgery^19,20^ and earlier work in the LASER study^8^. The rationale for this discrepancy is unclear; one possibility is a difference between studies in the retrospective *versus* contemporaneous nature of the questionnaire and any possible recall bias the former may suffer from. Regardless, the finding that pain at the thoracic incision site was not independently predictive of poor HRQoL in the present study is supported by the finding here that patients who had an open chest procedure were less likely than those with robotic or thoracoscopic procedures to have poor HRQoL, a finding reflected in studies comparing open thoracotomy and video-assisted thoracoscopic surgery in lung cancer surgery^21^. Heartburn was also not found to be independently predictive of poor HRQoL. However, this symptom was added to the LASORS tool after consultation with relevant patient advocacy groups and is one of the most prevalent post-oesophagectomy symptoms^8,22^ and thus likely remains of importance within the patient population despite its lack of statistical correlation with HRQoL alone.

It is important to consider the strengths and limitations of the study. In terms of strengths, the study recruited through the AUGIS research network from 15 tertiary UK centres with surgery performed between 2015 and 2019, so is representative of modern multimodal approaches to treatment of oesophageal cancer and is likely to have external validity within Europe. Development of the LASORS tool built on previous work from the LASER study, in combination with symptoms from a patient advisory group consultation process, conferring high relevance of symptoms to the wider oesophageal cancer patient population. Patients completed the LASORS, EORTC QLQ-C30, and EORTC QLQ-OG25 questionnaires in their own homes to avoid any bias being introduced by direct involvement of the clinical team in data collection. The LASORS tool evaluates symptoms contemporaneously, rather than over a retrospective time interval, removing recall bias.

The limitations of this study include its cross-sectional design, meaning the baseline symptoms and HRQoL of patients are unknown, and the longitudinal changes in symptoms and HRQoL were not studied. This study excluded non-disease-free survivors, which is in itself an important cohort of patients that requires specific investigation with regard to survivorship symptomatology and HRQoL. The study may also be subject to bias of well-motivated patients responding to the questionnaire. This study validated the LASORS tool in a UK population based on work previously carried out across Europe; there is a future need for further external validation of the LASORS tool in a non-European cohort.

The LASORS tool is a novel, clinically relevant, symptom-based tool that reflects HRQoL, negating the need to collect symptom and HRQoL data separately. Its benefit over existing tools, such as EORTC questionnaires, is two-fold. First, it is far less time-consuming and cumbersome, facilitating its real-time use in clinical practice. Second, it is specifically designed, and validated, for the disease-free post-treatment oesophageal cancer population (rather than those undergoing treatment); to the authors’ knowledge, it is the only such tool for this population.

The LASORS tool has multiple clinical uses. First, it provides a snapshot of the HRQoL of a patient and, when repeatedly administered throughout the follow-up interval, can provide a dynamic picture of a patient’s HRQoL. This aids in early identification of patients for whom HRQoL is poor or deteriorating, facilitating prompt intervention and mitigating the impact of symptoms on HRQoL. Second, the tool serves as a blueprint for establishing survivorship services and expertise, highlighting the symptoms of highest impact and relevance to the patient population. Survivorship services in oesophageal cancer are currently in their infancy^23,24^ and use of the LASORS tool over time, as patients engage with and benefit from early and tailored access to survivorship programmes, will provide evidence-based, validated data to support their ongoing development and funding. Third, use of the tool can relieve both financial and resource pressures from areas of the system not equipped to manage long-term post-treatment symptoms, redirecting these patients to tailored services, benefitting both the healthcare system and the patient experience. Finally, poor HRQoL can be defined by different thresholds and the performance of the LASORS tool, both in the context of combined poor function and symptoms, as well as one of these alone, highlights its utility and versatility in clinical practice.

## Supplementary Material

znae319_Supplementary_Data

## Data Availability

The data that support the findings of this study are available from the corresponding author upon reasonable request.
