# Supplementary material for: LAsting Symptoms after Oesophageal Resectional Surgery (LASORS): multicentre validation cohort study
Source: Br J Surg. 2025 Feb 21;112(2):znae319. doi: 10.1093/bjs/znae319 (PMC11843645; doi:10.1093/bjs/znae319)
Supplement: znae319_Supplementary_Data [file znae319_supplementary_data.docx]

Supplementary table 1: Number of patients per surgical centre

| **Surgical Centre** | **Patients** |
| --- | --- |
| Cambridge University Hospitals NHS foundation Trust | 43 |
| Oxford University Hospitals NHS Trust | 40 |
| Imperial College Healthcare NHS Trust | 34 |
| Leeds Teaching Hospitals NHS Trust | 34 |
| Nottingham University Hospitals NHS Trust | 32 |
| Salford Royal NHS Foundation Trust | 26 |
| Portsmouth Hospitals NHS Trust | 22 |
| University Hospitals Birmingham NHS Foundation Trust | 20 |
| University Hospitals Coventry and Warwickshire NHS Trust | 12 |
| South Tees Hospitals NHS Foundation Trust | 1 |
